# Supplementary figures and images for: Teasing apart the host-related, nutrient-related and temperature-related effects shaping the phenology and microbiome of the tropical seagrass Halophila stipulacea
Source: Environ Microbiome. 2022 Apr 15;17:18. doi: 10.1186/s40793-022-00412-6 (PMC9013022; doi:10.1186/s40793-022-00412-6)

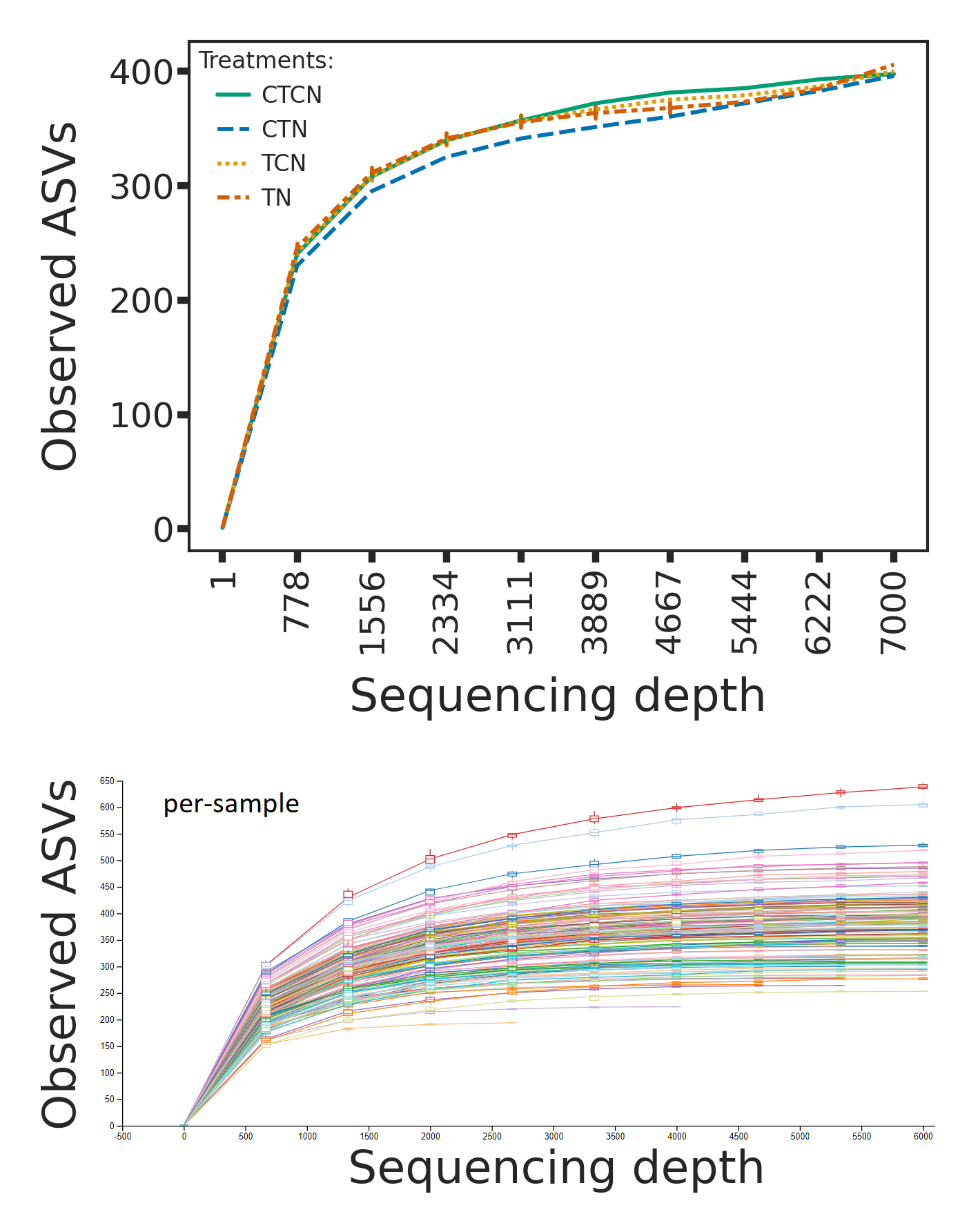

Supplement: Supplementary file 1 — Additional file 1. Figure S1: Alpha rarefaction curves of epiphytic microbial communities in samples of each mesocosm treatment [file 40793_2022_412_MOESM1_ESM.png]

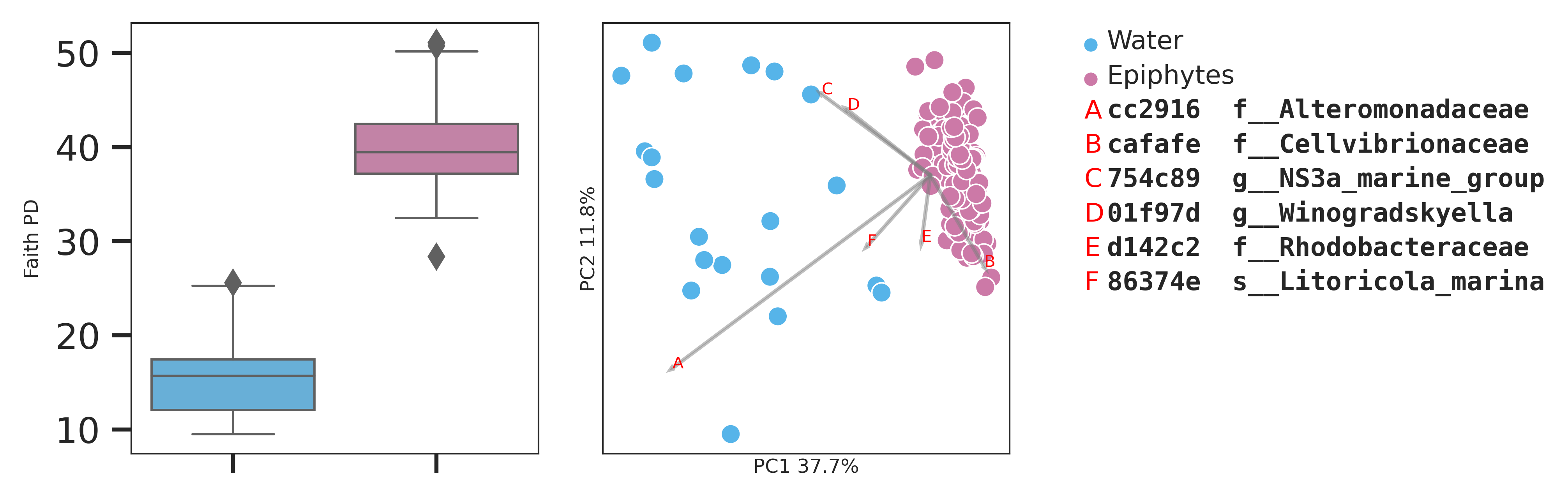

Supplement: Supplementary file 2 — Additional file 2. Figure S2: Alpha and beta diversity of mesocosm epiphyte and water samples. Faith’s phylogenetic diversity distributions in water and epiphyte samples are presented as a box plot. Weighted Unifrac distance based PCoA and Biplot are presented as ordination of PC1 and PC2 [file 40793_2022_412_MOESM2_ESM.tiff]

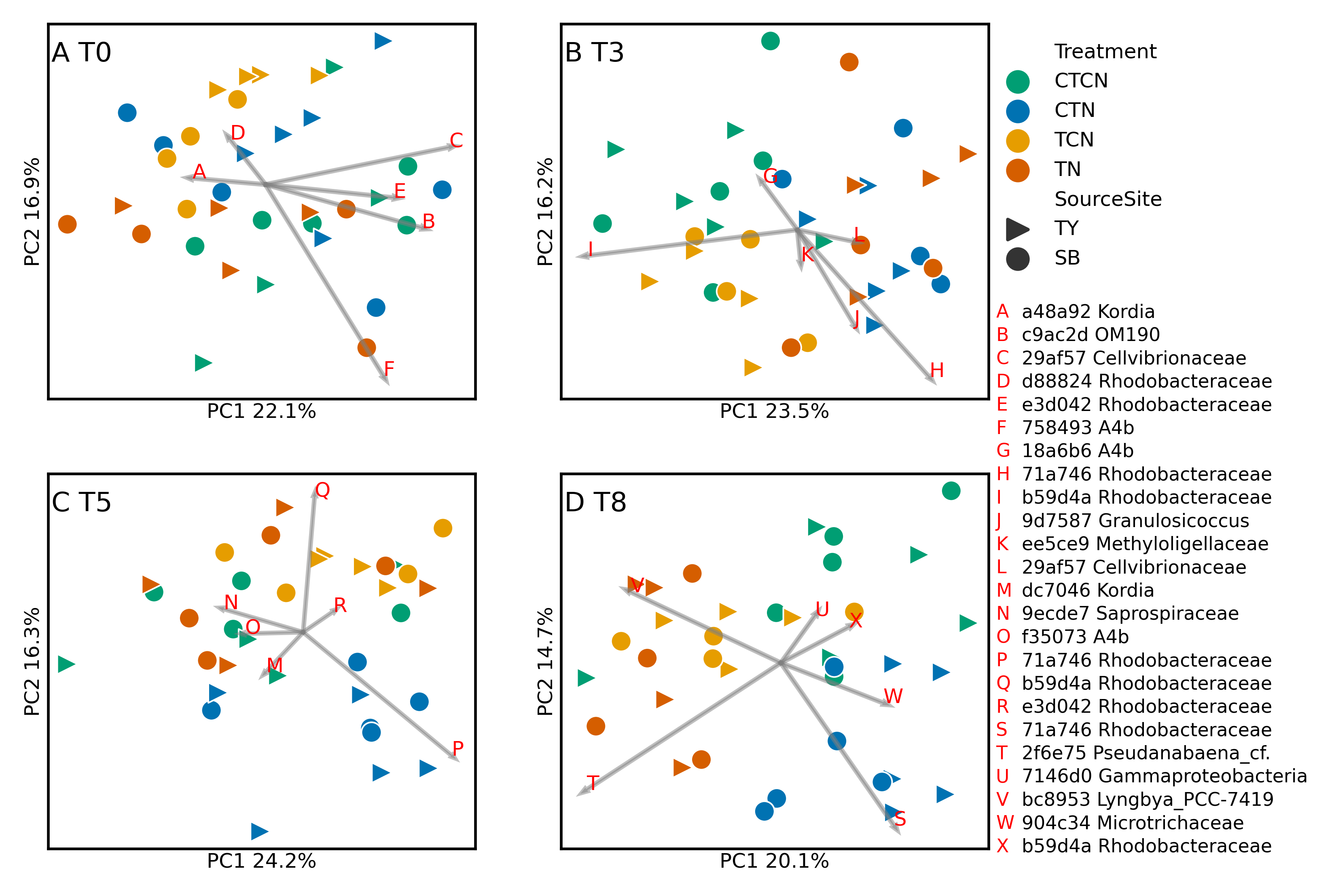

Supplement: Supplementary file 3 — Additional file 3. Figure S3: Diverging community compositions among treatments. Weighted UniFrac distance based principal coordinate analyses (PCoA) of epiphyte samples from T0 (A), T3 (B), T5 (C) and T8 (D). CTCN - control temperatures (27°C) and control nutrients (no enrichment). CTN - control temperatures (27°C) with nutrient enrichment. TCN - heatwave (31°C) without nutrient enrichment. TN - heatwave with nutrient enrichment. Time points T0 and T8 had baseline temperatures and no active nutrient enrichment in all baths. The percent total variance accounted for by each coordinate is indicated on the corresponding axis. The most important ASVs, following the importance definition by Legendre and Legendre [41], are represented by BiPlot analyses (gray arrows) and their taxonomic identifications are noted in the legend [file 40793_2022_412_MOESM3_ESM.png]

# Ancom Orders

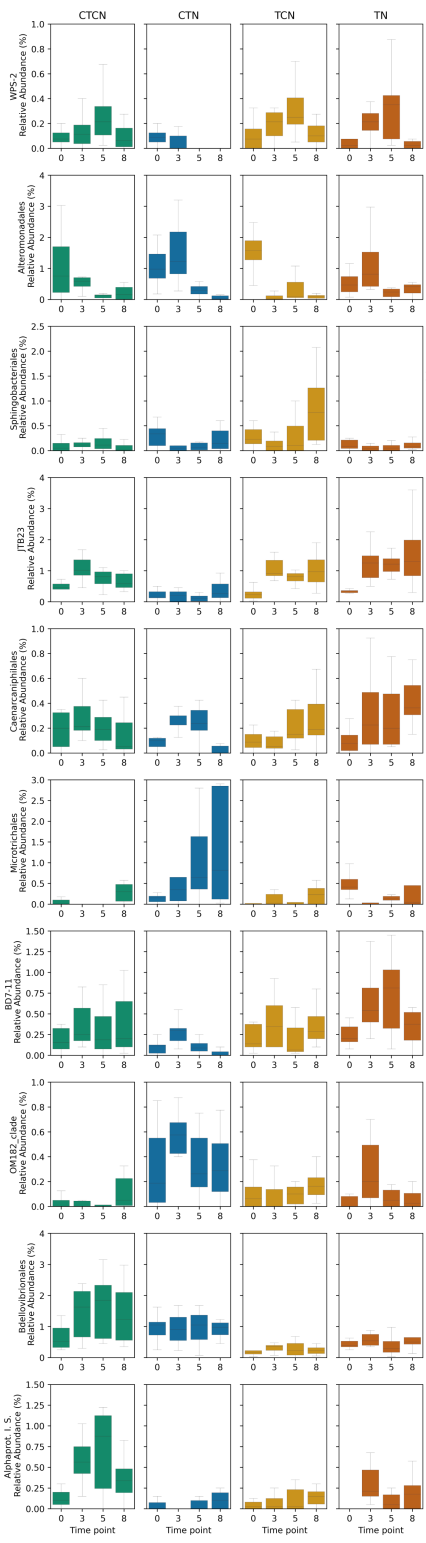

# Ancom Families

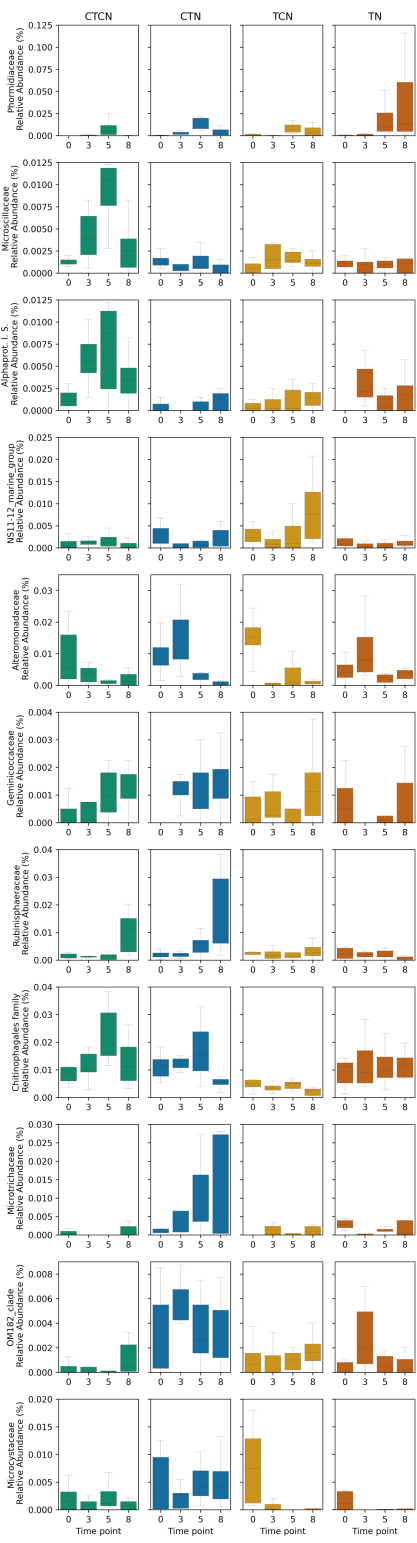

Supplement: Supplementary file 4 — Additional file 4. Figure S4: Family level dynamics of ANCOM families. CTCN - control temperatures (27°C) and control nutrients (no enriching). CTN - control temperatures with nutrient enriching. TCN - heatwave (31°C) without nutrient enriching. TN - heatwave with nutrient enriching. Timepoints T0 and T8 had baseline temperatures and no active nutrient enrichment in all treatments [file 40793_2022_412_MOESM4_ESM.pdf]

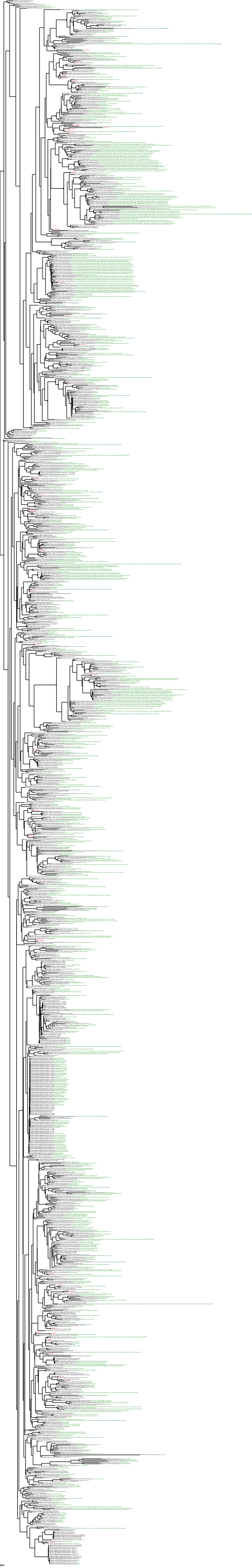

Supplement: Supplementary file 5 — Additional file 5. Figure S5: A phylogenetic tree of Rhodobacterales ASVs (red) along with reference sequences from the SILVA database (black) and their isolation source (green). Black bullets at the base of nodes represent a bootstrap percentage or 70 or higher [file 40793_2022_412_MOESM5_ESM.pdf]

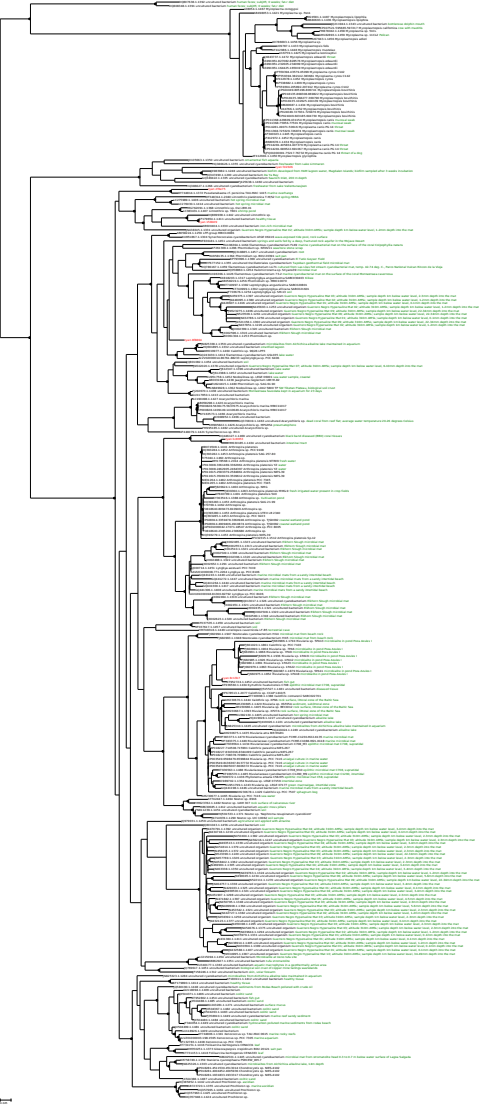

Supplement: Supplementary file 7 — Additional file 7. Figure S7: A phylogenetic tree of Cyanobacteria ASVs (red) along with reference sequences from the SILVA database (black) and their isolation source (green). Black bullets at the base of nodes represent a bootstrap percentage or 70 or higher [file 40793_2022_412_MOESM7_ESM.pdf]

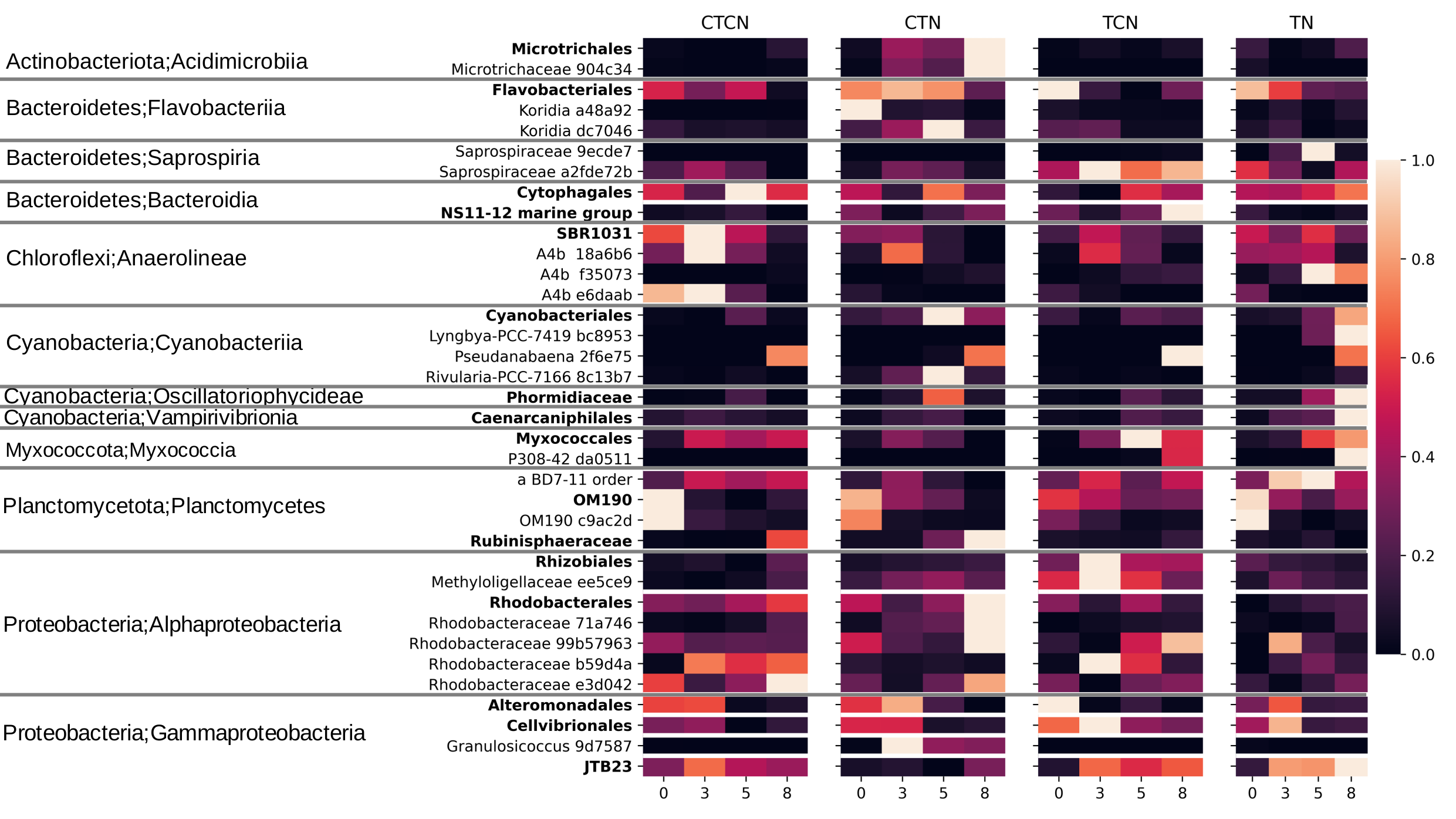

Supplement: Supplementary file 8 — Additional file 8. Figure S8: Dynamics of key features. Values are normalised by the peak relative abundance of each feature separately [file 40793_2022_412_MOESM8_ESM.tiff]

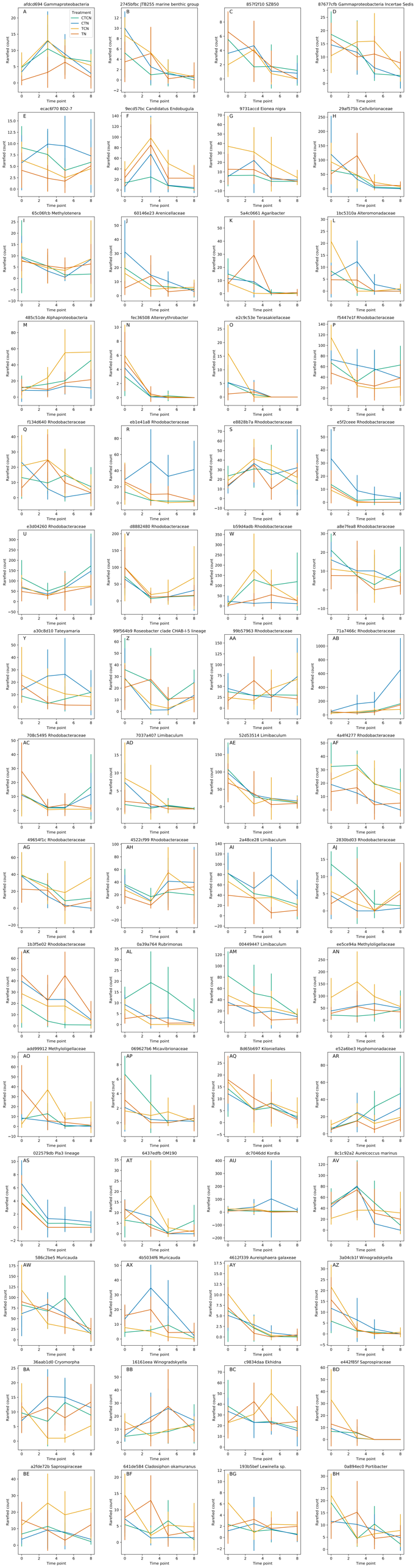

Supplement: Supplementary file 9 — Additional file 9. Figure S9: Relative abundance dynamics of core ASVs [file 40793_2022_412_MOESM9_ESM.pdf]

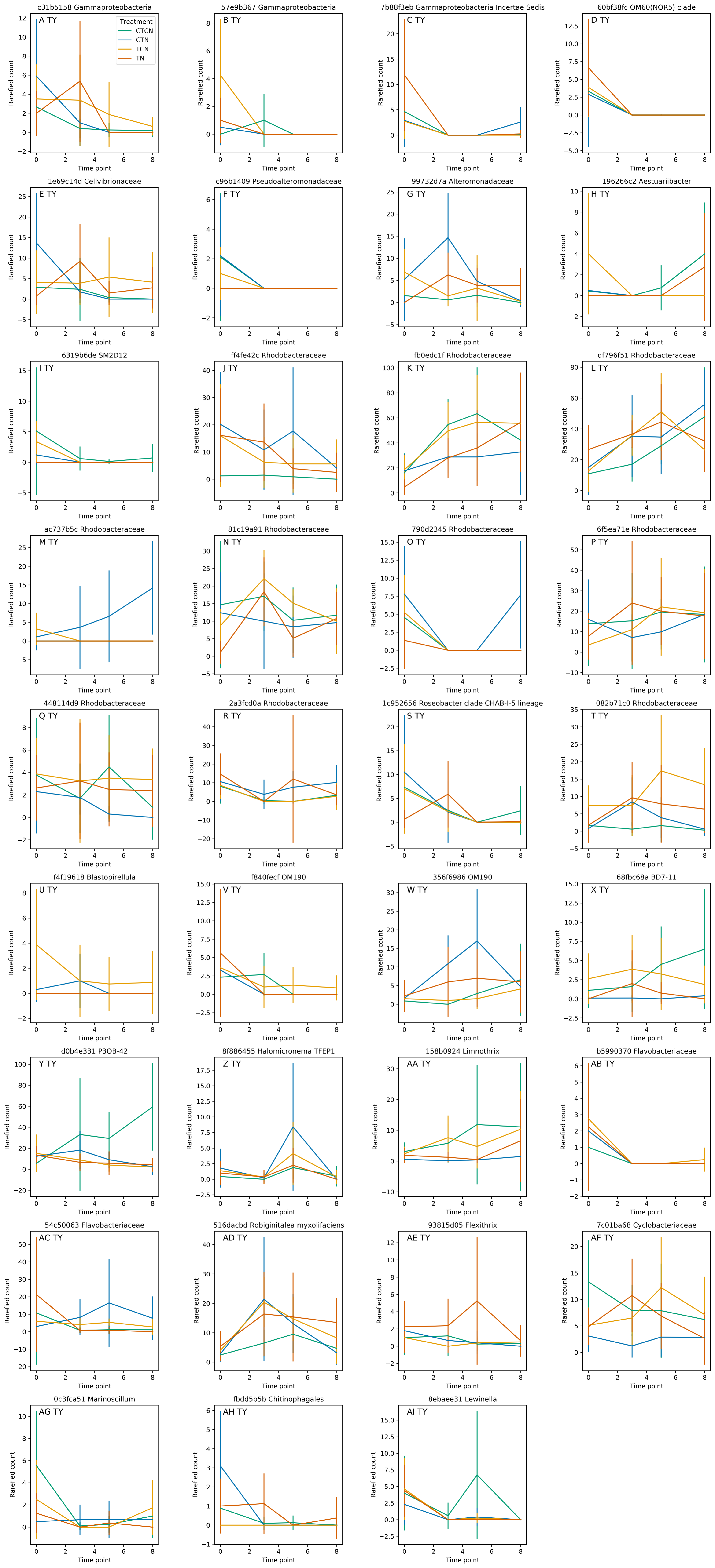

Supplement: Supplementary file 10 — Additional file 10. Figure S10: Relative abundance dynamics of TY core ASVs [file 40793_2022_412_MOESM10_ESM.pdf]

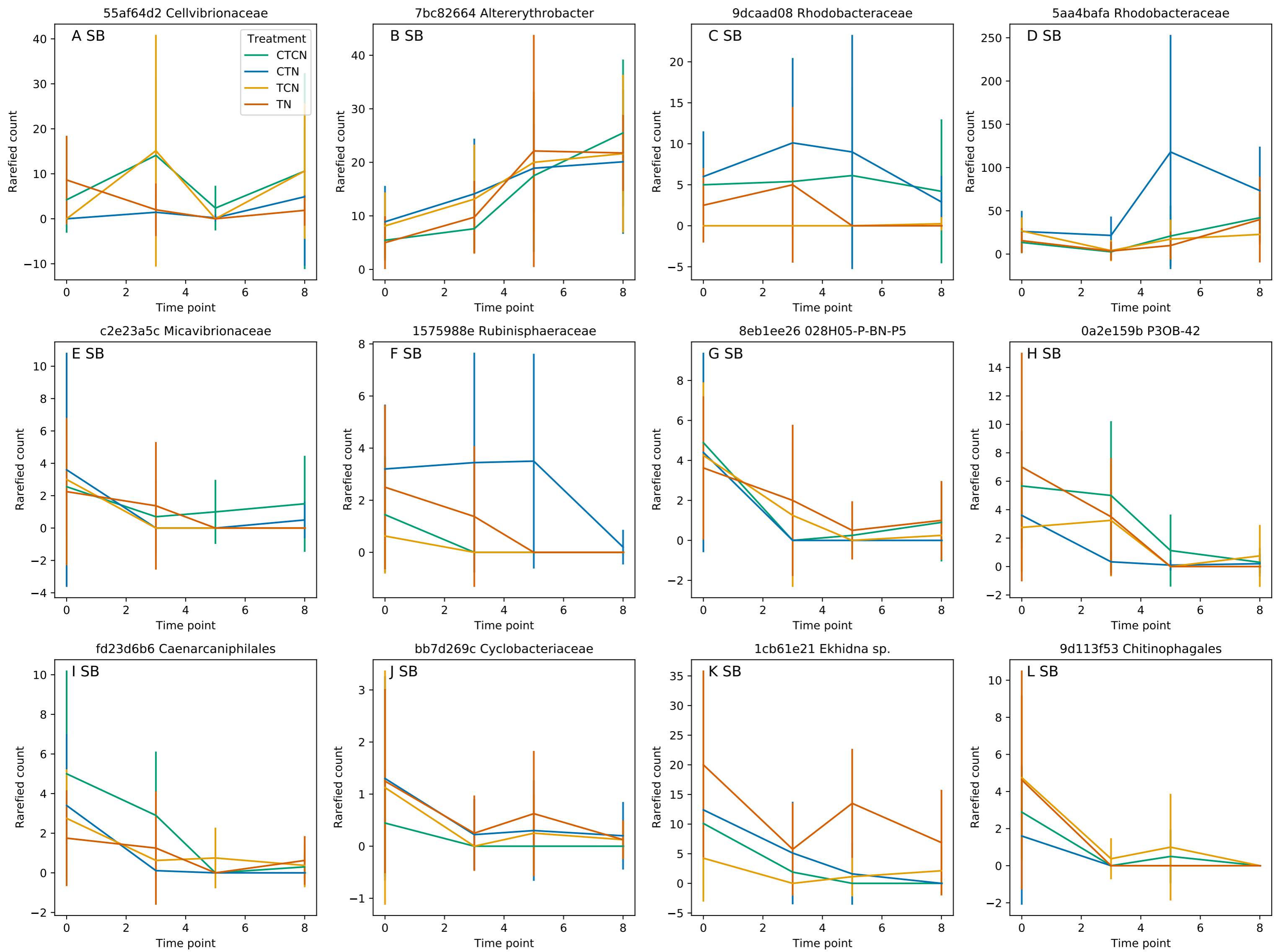

Supplement: Supplementary file 11 — Additional file 11. Figure S11: Relative abundance dynamics of SB core ASVs [file 40793_2022_412_MOESM11_ESM.pdf]
