# Supplementary material for: Teasing apart the host-related, nutrient-related and temperature-related effects shaping the phenology and microbiome of the tropical seagrass Halophila stipulacea
Source: Environ Microbiome. 2022 Apr 15;17:18. doi: 10.1186/s40793-022-00412-6 (PMC9013022; doi:10.1186/s40793-022-00412-6)
Supplement: Supplementary file 12 — Additional file 12. File S1: Alpha and beta diversity analyses of epiphytic community, without exclusion of ASVs that were shared with water samples [file 40793_2022_412_MOESM12_ESM.pdf]

File S1: Alpha and beta diversity analyses of epiphytic community, without exclusion of ASVs that were shared with water samples.

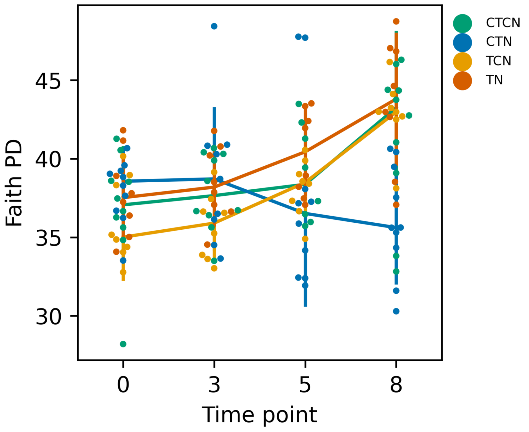

File S1, Fig. 1. Faith PD temporal dynamics. The four treatments (CTCN - control temperatures (27°C) and control nutrients (no nutrient enrichment). CTN - control temperatures with nutrient enrichment. TCN - heatwave (31°C) without nutrient enrichment. TN - heatwave with nutrient enrichment) are color coded. Line-plots represent the median Faith PD values and the bars denote standard deviations. Time point T0 and T8 had baseline conditions in all baths.

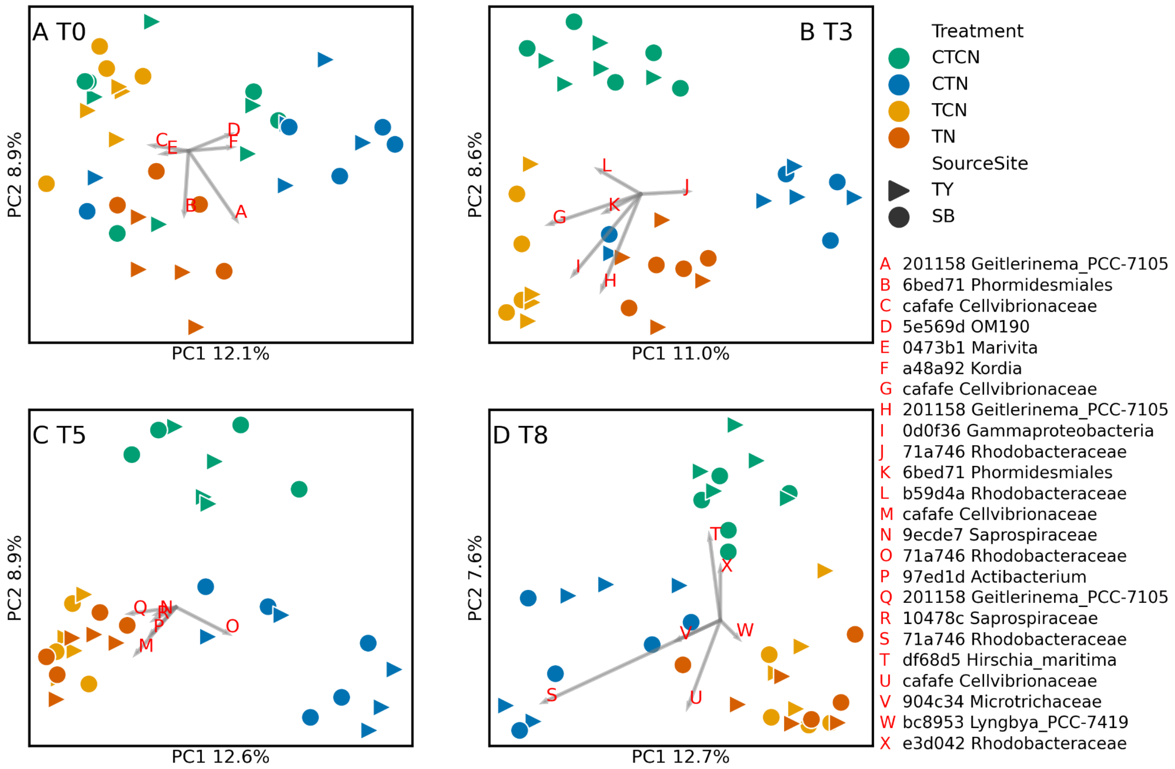

File S1, Fig. 2: Diverging community compositions among treatments. Unweighted UniFrac distance based principal coordinate analyses (PCoA) of epiphyte samples from T0 (A) ,T3 (B), T5 (C) and T8 (D). CTCN - control temperatures (27°C) and control nutrients (no enrichment). CTN - control temperatures (27°C) with nutrient enrichment. TCN - heatwave (31°C) without nutrient enrichment. TN - heatwave with nutrient enrichment. Time points T0 and T8 had baseline temperatures and no active nutrient enrichment in all baths. The percent total variance accounted for by each coordinate is indicated on the corresponding axis. The most important ASVs, following the importance definition by Legendre and

Legendre [41], are represented by BiPlot analyses (gray arrows) and their taxonomic identifications are noted in the legend.

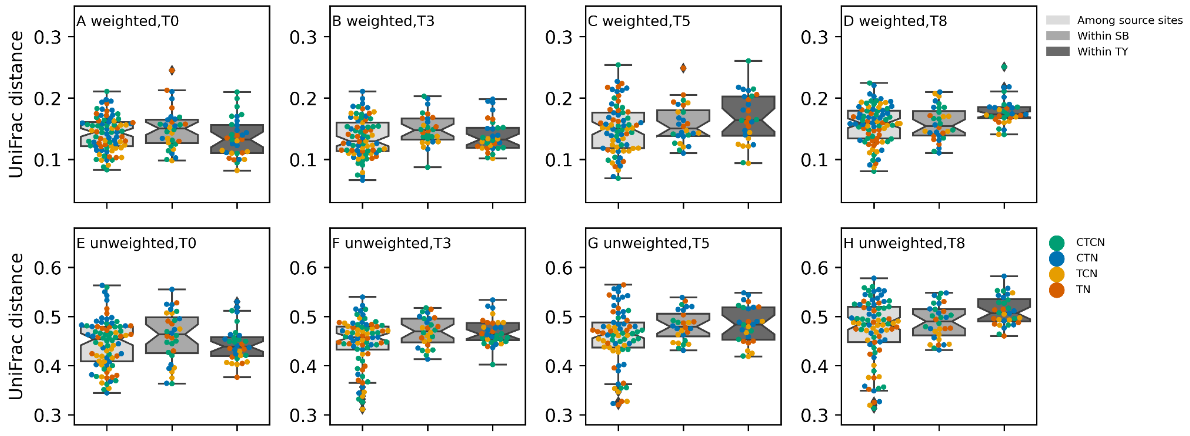

File S1, Fig. 3: Dynamics of UniFrac pairwise distances within and among source sites along the experimental time frame. The distributions of among-site, within SB and within TY distances are presented as box plots (see legend), using weighted (A-D) and unweighted (E-H) distances. Time points 0 (A & E), 3 (B & F), 5 (C & G) and 8 (D & H) are presented separately. The swarm plots reflect the distribution of pairwise distances among the different treatments.
